# Are the General Medical Council’s Tests of Competence fair to long standing doctors? A retrospective cohort study

**DOI:** 10.1186/s12909-015-0362-x

**Published:** 2015-04-21

**Authors:** Leila Mehdizadeh, Alison Sturrock, Jane Dacre

**Affiliations:** 1 Division of UCL Medical School, University College London, Royal Free Hospital, room GF/664, Hampstead, London, NW3 2PF UK; 2 Royal College of Physicians, London, UK

**Keywords:** General medical council, Fitness to practise, Tests of competence, Volunteer, Pilot, Assessment, Performance, Qualification year

## Abstract

**Background:**

The General Medical Council’s Fitness to Practise investigations may involve a test of competence for doctors with performance concerns. Concern has been raised about the suitability of the test format for doctors who qualified before the introduction of Single Best Answer and Objective Structured Clinical Examination assessments, both of which form the test of competence. This study explored whether the examination formats used in the tests of competence are fair to long standing doctors who have undergone fitness to practise investigation.

**Methods:**

A retrospective cohort design was used to determine an association between year of primary medical qualification and doctors’ test of competence performance. Performance of 95 general practitioners under investigation was compared with a group of 376 volunteer doctors. We analysed performance on knowledge test, OSCE overall, and three individual OSCE stations using Spearman’s correlation and regression models.

**Results:**

Doctors under investigation performed worse on all test outcomes compared to the comparison group. Qualification year correlated positively with performance on all outcomes except for physical examination (e.g. knowledge test r = 0.48, p < 0.001 and OSCE r = 0.37, p < 0.001). Qualification year was associated with test performance in doctors under investigation even when controlling for sex, ethnicity and qualification region. Regression analyses showed that qualification year was associated with knowledge test, OSCE and communication skills performance of doctors under investigation when other variables were controlled for. Among volunteer doctors this was not the case and their performance was more strongly related to where they qualified and their ethnic background. Furthermore, volunteer doctors who qualified before the introduction of Single Best Answer and OSCE assessments, still outperformed their peers under investigation.

**Conclusions:**

Earlier graduates under fitness to practise investigation performed less well on the test of competence than their more recently qualified peers under investigation. The performance of the comparator group tended to stay consistent irrespective of year qualified. Our results suggest that the test format does not disadvantage early qualified doctors. We discuss findings in relation to the GMC’s fitness to practise procedures and suggest alternative explanations for the poorer performance of long standing doctors under investigation.

## Background

The perception of fairness in clinical examinations in the UK came under scrutiny recently when an aspect of the Membership of the Royal College of General Practitioners examination was independently reviewed. International medical graduates and well trained UK ethnic minority doctors were found to consistently have significantly higher rates of failure on this examination than white UK trained doctors [1-3]. In the document Standards for Educational and Psychological Testing “fairness” in assessment can be defined in multiple ways [4]. In this article we draw upon the definition that a fair test is one that is free of bias, in that it is not associated with systematically higher or lower scores for identifiable groups of examinees [4]. Our concerns were about whether a high stakes examination used by the UK’s medical regulatory body was fair to all of the doctors who took it, given that this group of doctors is diverse in terms of ethnicity, age, place and year of qualification. In particular we were interested in whether doctors’ year of qualification was associated with differences in performance on a high stakes clinical examination.

The General Medical Council (GMC) holds a right to investigate the fitness to practise of doctors working in the UK [5]. In 2013 the GMC received 10, 013 enquiries about doctors, of which 80% were complaints about concerns with fitness to practise [6]. Most of these complaints were made by the public, 12% from other doctors, 6% from employers and 17% from other sources [6]. The GMC triage these complaints and decide whether to launch an investigation or not. At the broadest level, allegations about fitness to practise are categorised into health, conduct and/or performance related issues [6].

Where concerns are raised in relation to a doctor’s professional performance, a performance assessment may be carried out. A few examples of the concerns that may trigger an assessment of a doctor’s performance include a basic lack of knowledge/poor clinical judgement, inappropriate prescribing, tendency to use outdated techniques, poor record keeping and communication problems with patients and/or colleagues. The performance assessment consists of two parts: peer review and test of competence [7,8]. The test of competence is used to identify potential gaps in a doctor’s knowledge base and/or their clinical skills. This includes a written knowledge test using Single Best Answer (SBA) format and an Objective Structured Clinical Examination (OSCE) of which both parts are closely tailored to the doctor’s grade, speciality and clinical work. There is no ‘pass mark’ for these tests; instead the marks achieved by the doctor under investigation are compared to the range of marks achieved by a comparison group of doctors who have volunteered to take a similar test in the same specialty. Trained investigators at the GMC use all of the above aspects of a performance assessment to reach a judgement about a doctor’s fitness to practise. The most serious cases go on to be reviewed in a quasi-court hearing where final decisions are made.

The GMC’s tests of competence have gained worldwide recognition as valid and reliable assessment tools of clinical performance [9]. They are designed to test whether a doctor is performing at a minimum level of competence that is expected of a junior doctor two years post-graduation. The items included in each test are rigorously evaluated to ensure the tests are fair and fit for purpose before being used in fitness to practise investigations. This is achieved through regular piloting events organised and delivered by University College London (UCL) Medical School. Doctors with no known fitness to practise concerns are recruited by UCL Medical School to voluntarily take a test of competence in their relevant specialty. While these pilot examinations are similar to the tests the GMC implement in their investigations, the main difference is the extent to which the tests are tailored to doctors’ clinical practice. In fitness to practise investigations, the test of competence is tailored to reflect each doctor at an individual level according to their demographics and the clinical work they currently engage in. They are not examined on any aspect of clinical work that is not relevant to them at the time of investigation. For the purpose of evaluating the test items, pilot examinations are tailored to a group of doctors working in the same specialty rather than at the individual level. As well as evaluating the test items, the pilot events collect reference data by specialty that is used to compare with the performance of a doctor undergoing fitness to practise investigation.

The GMC’s tests of competence are regularly reviewed to ensure they conform to current best pedagogy. Consequently, the test format has altered several times and currently consists of a machine marked knowledge test of SBA items and an OSCE. As the average age of doctors having a performance assessment is 55, it is possible that these doctors will be unfamiliar with SBAs or OSCE examinations. There is evidence that test preparation is linked to improved performance and test familiarity has also been found to correlate positively with test performance [10-12]. Concern has been expressed as to whether the GMC’s test of competence format is fair for doctors who graduated before the SBA and OSCE format were in common use in medical education. We investigated this perception by studying whether there was an association between year of primary medical qualification and performance in a test of competence. Specifically the research questions were;

1. Is there a relationship between qualification year and test performance in doctors undergoing fitness to practise investigation?
2. Is there a relationship between qualification year and test of competence performance in volunteer doctors with no known concerns?
3. Is there a relationship between qualification year and test of competence performance when controlling for sex, ethnicity and region of qualification in both groups of doctors?

## Methods

This was a retrospective cohort study, with two groups. The first group (the investigation group) was composed of 95 general practitioners who took a test of competence as part of a GMC investigation between July 2009 and February 2013. The second group (the volunteer group) was composed of 365 doctors who volunteered to take a test of competence in general practice between January 2009 and March 2013. Volunteer doctors had no known fitness to practise concerns and explicitly consented to their data being used anonymously for research purposes. Consent was not required from doctors under investigation and this was approved by UCL Research Ethics Committee.

For practical reasons, we chose to analyse data from doctors who had taken a test in general practice as it represented our largest data set. The test of competence was a 120 item SBA knowledge test and a 12 station OSCE. The knowledge test was machine marked and scored out of 120. OSCE stations were marked by trained experienced general practitioners using the categories ‘acceptable’, ‘cause for concern’ and ‘unacceptable’. Each OSCE station was scored out of 40, and doctors could receive a maximum score of 480. We compared the performance of both groups using five outcome measures; 1) knowledge test score, 2) overall OSCE score, 3) OSCE Basic Life Support score, 4) OSCE communication skills score, 5) OSCE physical examination score. There were four independent variables; sex, ethnicity, qualification year and qualification region. Communication was most commonly assessed using a “pre-pregnancy advice in an overweight woman” or a “post-menopausal bleeding via an interpreter” scenario. Physical examination skills were tested either through a scenario on examining a pregnant abdomen or of an injured elbow.

We compared the knowledge test and OSCE scores of both groups of doctors using Statistical Package for the Social Sciences version 21. Results were analysed in two phases. Descriptive statistics, t-tests and correlations were used to analyse data from 95 general practitioners under investigation and 376 volunteer doctors. Multiple regression analysis was conducted on the same cohorts of doctors except with fewer volunteer doctors for the purpose of matching the groups more closely based on qualification year. The regression models included sex, ethnicity, qualification year and qualification region as the independent variables and exam scores as the dependent variables. Qualification region and ethnicity variables were dichotomised and coded into UK versus non-UK and white vs non-white respectively. We conducted a hierarchical multiple regression that tested for a relationship between year doctors gained their primary medical qualification and exam scores.

We received written confirmation of ethics approval from UCL’s Research Ethics Committee in November 2009.

## Results

### Demographics

The demographics of general practitioners in this study as well as those on the General Practitioners Register [13] are summarised in Table 1. Doctors who were under fitness to practise investigation were more likely to be men, to be Asian and/or to have qualified overseas. Year qualified ranged from 1961 to 2003 but the majority of doctors qualified before 1980.

**Table 1:** **Demographic characteristics of general practitioners in this study as compared to the general practitioners register**

	Doctors under investigation (n = 95)	Volunteer doctors (n = 376)	Total on general practitioners register (n = 63,778)
**Sex**			
Male	79 (83%)	169 (45%)	32, 719 (51%)
Female	16 (17%)	207 (55%)	31, 059 (49%)
**Qualification region**			
United Kingdom	34 (36%)	324 (86%)	49, 198 (77%)
European Economic Area	7 (7%)	4 (1%)	3, 998 (6%)
International	54 (57%)	48 (13%)	10, 582 (17%)
**Ethnicity**			
White	27 (28%)	220 (59%)	
Black	17 (18%)	16 (4%)	
Asian	39 (41%)	112 (30%)	
Mixed	0	8 (2%)	
Other	12 (13%)	18 (5%)	
Not stated	0	2 (<1%)	
**Year of qualification**			
1961-1992	85 (89%)	69 (18%)	
1993-2005	10 (11%)	146 (39%)	
2006-2011	0	161 (43%)	

Doctors in the volunteer group were mostly white UK graduates and there were more women than men. Year qualified ranged from 1971 to 2011 and the majority of volunteers qualified after the year 2000. We also make a distinction between ‘early qualified’ and ‘recently qualified’ doctors in this article. One third of doctors in our sample achieved their primary medical qualification by the year 1992 and are referred to as ‘early qualified’ or ‘long standing’ doctors. Another third who achieved their primary medical qualification in 2006 or thereafter are referred to as ‘recently qualified’.

### Test of competence performance

**Figure 1:**
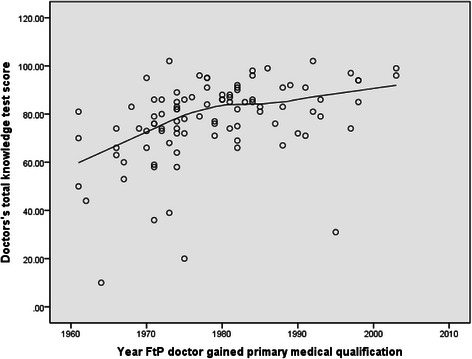
Scatter plot of knowledge test score against year of qualification in fitness to practise doctors under investigation.

**Figure 2:**
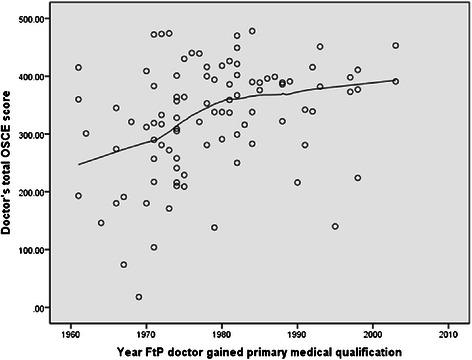
Scatter plot of total OSCE score against year of qualification in fitness to practice doctors under investigation.

**Figure 3:**
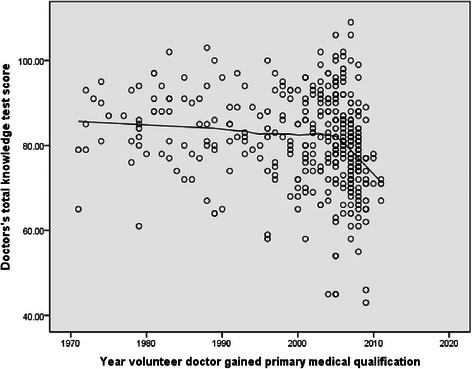
Scatter plot of knowledge test score against year of qualification in volunteer doctors.

**Figure 4:**
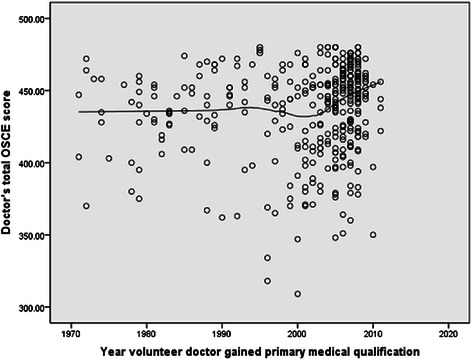
Scatter plot of total OSCE score against year of qualification in volunteer doctors.

Doctors in the fitness to practise group on average scored less than doctors in the volunteer group on all outcome measures (Table 2). In the fitness to practise group, doctors who had been qualified longest scored less in the knowledge test (Figure 1), the OSCE overall (Figure 2) basic life support and communication skills stations. Volunteer doctors remained fairly consistent in knowledge test (Figure 3) and OSCE performance (Figure 4) irrespective of their year of qualification.

**Table 2:** **Means and standard deviations of test performance in doctors under investigation compared to volunteer doctors**

	Mean score knowledge test	Mean score overall OSCE	Mean score basic life support	Mean score communication	Mean score physical examination
Doctors under investigation (n = 95)	77(SD = 16.9)	327 (SD = 96.8)	22(SD = 14.7)	27(SD = 13)	27(SD = 13.3)
Volunteer doctors (n = 376)	80(SD = 10.9)	437 (SD = 31.7)	35(SD = 6.5)	36(SD = 6.8)	37(SD = 5.4)
T-test	t(469) = −2.3, p = 0.02	t(469) = 18.5, p < 0.001	t(469) = −13.6, p < 0.001	t(469) = −9.6, p < 0.001	t(469) = −12.1, p < 0.001
Standardized mean differences	Cohen’s d = −0.21	Cohen’s d = −1.53	Cohen’s d = −1.14	Cohen’s d = −0.87	Cohen’s d = −0.99
	Effect size r = −0.1	Effect size r = −0.61	Effect size r = −0.5	Effect size r = −0.4	Effect size r = −0.44

### Knowledge test performance

There was a significant positive relationship between year of qualification and knowledge test score in doctors under investigation (r = 0.48, p < 0.001) (Figure 1). There was no relationship between knowledge test performance and year of qualification in the volunteer group. Knowledge test performance in this group remained fairly consistent irrespective of qualification year until the year 2000 (Figure 3). Volunteer doctors who qualified after the year 2000 scored less in the knowledge test (r = −0.29, p <0.001).

### OSCE performance

#### *Overall OSCE score*

In the fitness to practise group there was a moderate positive association between year of qualification and overall OSCE score (r = 0.37, p < 0.001), with doctors who had been qualified longest scoring less than more recent graduates (Figure 2). In the volunteer group, there was a weaker association between year of qualification and overall OSCE score (r = 0.2, p = <0.001). OSCE performance remained fairly consistent until around the year 2000. Doctors who qualified after 2000 scored higher in overall OSCE performance (Figure 4).

#### *Basic life support score*

In the fitness to practise group there was a significant positive association between year of qualification and their basic life support score (r = 0.28, p = 0.006) with doctors who had been qualified longest scoring less than more recent graduates. In the volunteer group there was no correlation between year of qualification and score on basic life support (r = 0.03, p = 0.62).

#### *Communication skills score*

In the fitness to practise group there was a significant positive correlation between year of qualification and communication station score (r = 0.3, p = 0.001) with doctors who had been qualified longest scoring less than more recent graduates. This was also seen in the volunteer group but the positive relationship was weaker (r = 0.2, p < 0.001).

#### *Physical examination score*

There was no association between year of qualification and physical examination score in either group (r = 0.08, p = 0.43: r = 0.09, p = 0.1).

### Multiple regression analysis

We matched the two groups more closely based on qualification year to conduct a multiple regression analysis by removing volunteer doctors from the sample who had qualified after the year 2003 (n = 159). Year 2003 was used as a cut-off point as none of the doctors under investigation (n = 95) had received their primary medical qualification after this date. The results are summarised in Tables three to seven where sex, ethnicity, qualification year and qualification region were the independent variables and exam scores were the dependent variables. In model one we included the independent variables sex, ethnicity and qualification region, followed by qualification year in model two. As the two groups differ by sex, ethnicity and region of qualification (Table 3) we conducted separate regression analysis for investigated and volunteer doctors to facilitate interpretation of the results.

**Table 3:** **Demographics of both groups of doctors who qualified between years 1960-2003**

	Doctors under investigation (n = 95)	Volunteer doctors (n = 159)
Sex		
Male	79 (83%)	88 (55%)
Female	16 (17%)	71 (45%)
Ethnicity		
White	26 (27%)	95 (60%)
Non-white	69 (73%)	64 (40%)
Region of qualification		
UK	34 (36%)	116 (73%)
Overseas	61 (64%)	43 (27%)
Year of qualification		
1960-1980	56 (59%)	22 (14%)
1981-1990	25 (26%)	39 (25%)
1991-2003	14 (15%)	98 (62%)

#### Knowledge test performance

Among doctors under investigation, a significant proportion of the variance in knowledge test performance was explained by adding qualification year to the model (22%) (Table 4). The main effect of each model was also significant indicating there is a relationship between the independent variables and knowledge test performance. Qualification region was significantly associated with knowledge test performance, with non-UK graduates scoring on average 9.8 marks lower than UK graduates. There was also a significant relationship between qualification year and knowledge test performance; for every one unit increase in qualification year, doctors performed on average 0.6 of a mark better on the knowledge test.

**Table 4:** **Multiple regression results for knowledge test performance in doctors under investigation**

Regression in FtP	R	Adjusted R square	R square change	Sig F change	ANOVA	Coefficients
Model 1*	0.4	0.14	0.16	P = 0.001	F(3,94) = 5.95, p = 0.001	PMQ region B = −11.82, Beta = − 0.34, p = 0.008
Model 2**	0.51	0.22	0.09	P = 0.001	F(4,94) = 7.70, p < 0.001	PMQ region B = −9.78, Beta = −0.28, p = 0.001
						PMQ year B = 0.57, Beta = 0.32, p = 0.001

*Model 1 variables: gender, ethnicity, qualification region.

**Model 2 variables: gender, ethnicity, qualification region, qualification year.

In the volunteer group, qualification year was not associated with knowledge test performance among volunteers (Table 5). However there was a relationship between region of qualification and knowledge test performance, with overseas trained doctors on average scoring 8.50 marks lower than UK graduates.

**Table 5:** **Multiple regression results for knowledge test performance in volunteer doctors**

Regression in volunteers	R	Adjusted R square	R square change	Sig F change	ANOVA	Coefficients
Model 1*	0.16	0.15	0.16	P < 0.001	F(3, 158) = 10.01, p < 0.001	PMQ region B −8.29, Beta −0.38, p < 0.001
Model 2**	0.17	0.15	0.01	P = 0.28	F(4, 158) = 7.81, P < 0.001	PMQ region B −8.50, Beta −0.38, p < 0.001

*Model 1 variables: gender, ethnicity, qualification region.

**Model 2 variables: gender, ethnicity, qualification region, qualification year.

#### Overall OSCE performance

Among doctors under investigation, the proportion of variance in OSCE performance increased from 13 to 17% when qualification year was added to the model (Table 6). There was a significant relationship between qualification year and OSCE performance; for every one unit increase in qualification year, doctors performed on average 2.4 of a mark better on the OSCE. There was no relationship between qualification year and OSCE performance among volunteer doctors (Table 7). Ethnicity was the single most variable that was associated with OSCE performance among investigated doctors, with white doctors scoring an average 67 marks higher than their non-white peers. Whereas significant relationships existed between the sex, ethnicity and qualification region of volunteer doctors and their OSCE performance. Women on average scored 11 marks higher than men (Table 7). White volunteer doctors scored on average 20.1 marks higher than non-white doctors. Qualification region had the strongest relationship with OSCE performance, with UK graduates scoring on average 23.17 marks higher than non-UK graduates (Table 7).

**Table 6:** **Multiple regression results for overall OSCE performance in doctors under investigation**

Regression in FtP	R	Adjusted R square	R square change	Sig F change	ANOVA	Coefficients
Model 1*	0.40	0.13	0.16	5.66	F(3,94) = 5.66, p = 0.001	Ethnicity B = 71.6, Beta = 0.33, p = 0.009
Model 2**	0.45	0.17	0.05	5.60	F(4,94) = 5.86, p < 0.001	Ethnicity B = 66.97, Beta = 0.31, p = 0.01
						PMQ year B = 2.4, Beta 0.24, p = 0.02

*Model 1 variables: gender, ethnicity, qualification region.

**Model 2 variables: gender, ethnicity, qualification region, qualification year.

**Table 7:** **Multiple regression results for overall OSCE performance in volunteer doctors**

Regression in volunteers	R	Adjusted R square	R square change	Sig F change	ANOVA	Coefficients
Model 1*	0.52	0.25	0.27	P < 0.001	F(3,158) = 18.80, p < 0.001	Gender B = −11.00, Beta −0.16, p = 0.025
						Ethnicity B = 16.54, Beta 0.23, p = 0.02
						PMQ region B = −22.11, Beta −0.29, p = 0.003
Model 2**	0.53	0.26	0.01	P = 0.1.9	F(4, 158) = 14.90, p < 0.001	Ethnicity B = 20.1, Beta 0.28, p = 0.005
						PMQ region B = −23.17, Beta −0.30, p = 0.002

*Model 1 variables: gender, ethnicity, qualification region.

**Model 2 variables: gender, ethnicity, qualification region, qualification year.

#### Basic Life support performance

None of the variables, including year of qualification, were associated with score on basic life support in either group.

#### Communication skills performance

Among the investigated group of doctors only a small proportion of variance in communication skills performance was accounted for by adding year of qualification into the regression model (7%). However there was still a significant relationship between qualification year and communication skills performance; as qualification year increases by one unit, performance increased by 0.34 of a mark. Qualification year was also associated with communication skills performance among volunteer doctors, with an increase of 0.23 of a mark for each unit of increase in qualification year. Ethnicity was also associated with their communication skills performance. White volunteer doctors performed on average 3.13 marks higher on the communication skills station than non-white volunteer doctors.

#### Physical examination performance

None of the variables, including year of qualification, were associated with physical examination performance in either group.

The relationship between doctors’ year of qualification and test of competence performance in both groups is summarised in Table 8.

**Table 8:** **Relationship of qualification year with each test outcome when controlling for sex, ethnicity and qualification region**

	Effect of qualification year in doctors under investigation	Effect of qualification year in volunteer group
Knowledge test	Early graduates perform worse than more recent graduates	No relationship, performance remains consistent
OSCE overall	Early graduates perform worse than more recent graduates	No relationship, performance remains consistent
Basic life support station	No relationship	No relationship
Communication skills station	Early graduates perform worse than more recent graduates	Early graduates perform well, more recent graduates perform even better
Physical examination station	No relationship	No relationship

## Discussion

### Summary of findings

On average, doctors under investigation performed worse on all aspects of the test of competence compared to volunteer doctors. Further, early graduates under investigation performed significantly poorer on the knowledge test and OSCE overall than their more recently qualified peers also under investigation. In the volunteer group, doctors tended to perform more consistently irrespective of year of qualification. Volunteer doctor who graduated after 2005 scored lower in the knowledge test but higher in overall OSCE performance and communication skills. The year of qualification was not associated with basic life support and physical examination performance in either group. Regression analyses showed that year of qualification was associated with knowledge test, OSCE and communication skills performance of doctors under investigation when other variable were controlled for. Among volunteer doctors this was not the case and their performance was more strongly related to where they qualified and their ethnic background.

### Findings in relation to literature

Our findings relate to a study carried out with doctors who were referred to an assessment programme in the USA due to concerns about their clinical competence [12]. Their performance on a test of patient management decreased as the age of the doctors increased. This pattern did not apply to the comparison group [12]. Poorer performance has also been found to be associated with increased age and/or year of qualification in doctors with no known performance concerns [14-16]. A systematic review also concluded that doctors who have been in practice longer may be at risk of providing lower quality care than more recently qualified peers [14]. Another study found an increased risk of post-operative complications in both inexperienced surgeons and those who had been practising longest [15].

In the volunteer group those who graduated in or after 2005 performed less well in the knowledge test. This most likely reflects a less sophisticated knowledge base due to less time in clinical practice. Doctors’ diagnostic accuracy has been found to be strongly positively associated with experience and this is thought to be linked to the development of pattern recognition as a doctor gains clinical experience [17-19]. In contrast, doctors in the volunteer group who qualified in or after 2005 scored higher in the overall OSCE. We do not know whether this same pattern applies to doctors under investigation as no one in this group had qualified after the year 2005.

We found evidence of a clear attainment gap by ethnic background and qualification region in both groups of doctors. A persistent attainment gap in clinical examinations has been reported between white and non-white medical students in the UK, USA and other English speaking countries. [20-22]. International medical graduates also perform less well on postgraduate medical examinations than UK graduates [1,23,24]. In this study, the majority of doctors under investigation were international medical graduates; they performed significantly worse on the knowledge test than UK graduates. OSCE performance was strongly associated with ethnic background. In the fitness to practise group, white doctors, who were a minority, performed significantly better in the OSCE overall than non-white doctors. Similarly in the volunteer group, white UK graduates outperformed their non-white overseas trained peers on the OSCE overall. The reason for this performance gap is unclear but cross-cultural differences in communication styles may explain some of the variation. Further research is necessary to understand why these ethnic differences persist in medicine [20,24].

### Implications

As the results from a test of competence are part of the process of judging whether a doctor is fit to practise, it is important to ensure that they are fair to all doctors. From this study, there is no clear evidence that long standing doctors under investigation are disadvantaged by the test of competence format. If this were the case, we would have expected to see a similar pattern among volunteer doctors and in particular those with comparable qualification years to doctors under investigation. Volunteer doctors who graduated in the last 10 years performed particularly well on the OSCE overall. Volunteer doctors who graduated over 10 years ago still performed well in the OSCE and also scored higher on the knowledge tests than more recent graduates. As all of the doctors under investigation graduated before 2003 we are unable to look for a similar pattern in this group. While doctors under investigation differed in their demographic profile compared to the volunteer group, the volunteer group were more similar to the General Practitioners Register in terms of sex and qualification region. We are currently reviewing how to increase the representativeness of our reference group data to reduce demographic differences between doctors who participate in piloting and the wider UK medical population [25].

Given these findings, we have to look to alternative explanations for the difference in performance between early and recent graduates among doctors under investigation, as volunteers remained largely consistent in their performance irrespective of when they qualified. One explanation may be that experience alone does not sufficiently maintain clinical knowledge and skills and that a doctor needs to make a conscious effort to remain up to date [6,26]. Doctors who have been qualified longest are thought to be less accepting of shifts in theoretical knowledge, best practices and/or medical technologies, and may be less likely to engage in regular continuing professional development [27,28]. This is also thought to be the case for poorly performing doctors who are less likely to remain up to date and more likely to work in isolation [29]. Doctors who engage with high quality continuing professional development activities have been found to demonstrate better clinical performance than those who do not [30].

### Limitations

The main limitation is that the demographics between the two cohorts are different. The majority in the volunteer group were recent graduates whereas the majority of the fitness to practise group graduated more than 30 years ago. It should however be noted that volunteer doctors who graduated between 1970 and 1992 still performed better on all outcomes than doctors in the fitness to practise group. The demographic differences exist because the data was not collected specifically for this study and we only analysed a sample of a large data set for practical reasons. Although we have data from volunteer doctors and investigated doctors working in a wide variety of hospital specialties, the patterns found in this study are only applicable to those who have taken a test in the specialty general practice. Therefore we do not know whether these results apply to doctors working in medical or surgical specialties. We also cannot comment on how the volunteer group compares to the wider population of general practitioners in the UK due to an absence of data on ethnicity and year of qualification. However it is important to note that the experts who are involved in making judgements and decisions about poorly performing doctors are aware of the limitations with the reference group data and take this into account. Doctors under investigation are not judged solely on their test of competence performance. Rather their performance is viewed in the wider context of a comprehensive peer review that provides in-depth information about their actual clinical practice. We have also reviewed the representativeness of the reference group in a separate research study [25]. As a result, we are tailoring our recruitment strategies to recruit more doctors from underrepresented groups to voluntarily pilot tests of competence [25].

## Conclusions

This is one of the first studies to explore whether doctors’ year of qualification is associated with performance in a high stakes examination. The large sample size means the study has sufficient power to detect differences between doctors under investigation and volunteer doctors. Our findings suggest that the current test of competence format does not disadvantage long standing doctors undergoing fitness to practise investigation. We found that when controlling for other variables, the year that doctors under investigation qualified was strongly associated with knowledge test and overall OSCE performance but less so on individual OSCE stations. More recently qualified doctors in the fitness to practise group performed better than earlier qualified peers. In the volunteer group, a weaker relationship was found between year of qualification and performance on knowledge test and OSCE. Factors that may have confounded the results include the unknown ages of doctors in both cohorts as well as their actual number of years in (and out of) clinical practice. Our results should be viewed as preliminary that require further study. Future work should look at whether there is a relationship between qualification year and test of competence performance when the age of doctors, years in clinical practice, and specialty are controlled for.
